# Strengths, Weaknesses, Opportunities, and Threats (SWOT) Analysis of Hemodialysis Electronic Health Record Implementation

**DOI:** 10.7759/cureus.54675

**Published:** 2024-02-22

**Authors:** Bassma Bennis, Ghita El Bardai, Basmat Amal Chouhani, Nadia Kabbali, Tarik Sqalli

**Affiliations:** 1 Laboratory of Epidemiology and Research in Health Sciences, Faculty of Medicine, Pharmacy and Dentistry, Sidi Mohamed Ben Abdellah University, Fez, MAR; 2 Nephrology Dialysis and Transplantation, Hassan II University Hospital, Fez, MAR

**Keywords:** hospital information system (his), swot (strengths weaknesses opportunities threats) analysis, electronic health record (ehr), software as a service (saas), electronic medical records (emr)

## Abstract

Background and aim: The Nephrology Department of Hassan II Hospital in Fez, Morocco, has implemented an Electronic Medical Record (EMR) system for managing patients undergoing acute hemodialysis. This initiative aims to digitize patient monitoring and enhance the management of acute dialysis within the department. Conducting strengths, weaknesses, opportunities, and threats (SWOT) analysis - assessing strengths, weaknesses, opportunities, and threats - was crucial to identifying and understanding the internal strengths and weaknesses, as well as the external opportunities and threats. This article outlines the SWOT analysis findings that may impact the project's success and shape decision-making. It also discusses strategies that could be implemented to allocate resources, mitigate risks, and capitalize on potential advantages.

Materials and methods: This study involved a multidisciplinary team, including professors, nephrologists, nephrology residents, and a healthcare information system engineer. Brainstorming sessions were held during the specification drafting phase to pinpoint both internal and external factors affecting the project. User feedback during testing further refined these factors, ensuring the project's alignment with real-world needs and challenges.

Results: The study identifies the project's strengths as providing safe and immediate access to information, along with strong communication between the department (application users) and the project manager. The significant EMR weakness is the lack of logistical resources and the absence of a long-term maintenance plan for the application. The opportunity presented by this EMR implementation is its functionality's potential to evolve, enabling the solution to be deployed in other dialysis centers across the region. The project's threat is the potential abandonment of EMR use by future practitioners.

Conclusion: These SWOT analysis findings enable the development and implementation of strategies to reduce the current deployment's vulnerabilities and ensure the success of future HIS implementations in the nephrology network of the Fez-Meknes region, Morocco.

## Introduction

Hemodialysis is one of the treatments for chronic renal failure. According to the Francophone Society of Nephrology, Dialysis, and Transplantation [1], hemodialysis allows the blood to be purified of toxins owing to an extracorporeal circuit between the patient's blood and dialysate solution. There are two main types of hemodialysis: chronic (long-term) hemodialysis and acute hemodialysis. The nephrology department of Hassan II University Hospital, Fez, Morocco, delivers acute hemodialysis sessions for patients needing hemodialysis treatment and hospitalization in the establishment. The department managed the acute hemodialysis activity through a paper register. Doctors recorded all the patient's prescriptions and medicines on the register. Consequently, it took a lot of work for physicians to follow up on patients who frequently returned to the service. Additionally, the data were complex, and no statistics were available to track the acute hemodialysis activity performances. The transition to a numeric register was essential for managing the activity better. As a result, the department decided to deploy a hemodialysis electronic medical record (EMR) named EKILI (E-Kidney in Arabic). Implementing a computerized medical record aims to provide physicians with digital access to all administrative and medical information about their patients. An EMR, or computerized medical record, is a health information system (HIS) that allows transversal patient data management in one practice. In addition, it enables practitioners to perform a medical follow-up on the patients through the data collected. The EMR provides quick and easy access to all stored information through an ergonomic interface. These data can be processed to analyze activity and care quality indicators. In addition, physicians can share EMR data to coordinate the medical pathway better. Access to and processing of EMR data must comply with the ethical requirements and legal framework for protecting personal data. In Morocco, according to "Law n°09-08" [2], any information relating to physical persons or mental health is considered confidential data. Therefore, a data processing authorization has been filed with the National Commission for the Control of Personal Data Protection (CNDP). There are many barriers to implementing and using EMRs. Therefore, a strengths, weaknesses, opportunities, and threats (SWOT) analysis was necessary to study the system implementation environment and identify its strengths and vulnerabilities. This article aims to present the SWOT analysis findings conducted throughout the deployment of the acute hemodialysis EMR in the nephrology department of Hassan II Hospital in Fez, Morocco.

## Materials and methods

Context

Hassan II University Hospital, Fez, Morocco's nephrology service, aims to care for all the region's patients with nephrological problems with or without renal failure. The structure receives patients in day hospitals, scheduled consultations, hospitalization, and in an emergency. It also ensures acute dialysis for all hospitalized patients needing hemodialysis treatment. Acute dialysis sessions must be prescribed in advance by an attending physician or a physician on the ward. The prescription provides the necessary dialysis parameters and the medical treatments to be performed during the session. In addition, acute dialysis requires patient monitoring by a nephrologist and a nurse. During the session, the physician regularly checks biological parameters such as weight, blood pressure, and temperature. In addition, he records all the medical acts performed. Thus, acute dialysis activity produces a significant amount of daily medical data. Therefore, it is necessary to set up a simple and efficient hemodialysis information system in the department.

EKILI

EKILI is an acute hemodialysis electronic medical record. It is a web application developed using software as a service (SAAS) technology. Its primary users are physicians in the nephrology department. The platform gathers acute hemodialysis patients' files. It contains patients’ administrative and medical data, prescriptions, and hemodialysis monitoring files. The physician creates a patient record for every new department hemodialysis service admission. Then, he makes and schedules a hemodialysis prescription for each patient by entering several prescription parameters. During the hemodialysis session, the doctor controls the patient's state by recording several physiological constants and vital signs in the hemodialysis monitoring file. Thus, EKILI digitally manages the acute hemodialysis activity. It tracks all procedures and archives hemodialysis session files. The objective of EKILI is to gradually replace the paper register of hemodialysis patients with a digital version. It can progressively transform medical practices and improve the organization of the daily workflow of the department's hemodialysis service. Furthermore, all parameters entered in EKILI are dynamic variables. Therefore, it is possible to produce different statistics to monitor the department's quality indicators of dialysis activity.

Type of study

The study aims to analyze the internal and external factors impacting the deployment of the hemodialysis Electronic Medical Record (EMR). In doing so, the department can devise profitable strategies for success in current and future health information system (HIS) implementations. This study was primarily based on a SWOT analysis of the indicators observed before and during the EMR implementation period. It was conducted by the Nephrology Department of Hassan II University Hospital in Fez, supported by a diverse team that included professors, nephrologists, residents, and a health information systems engineer. This team benefited from initial brainstorming sessions while drafting the specifications, which helped identify the factors influencing the project. Subsequently, regular meetings throughout the implementation phase consolidated the identification and analysis of these factors, further enriched by user feedback during the testing phases. Finally, the study included benchmarking HIS implementations in other countries, notably the US, Canada, India, Portugal, and Iran.

SWOT (Strengths, Weaknesses, Opportunities, Threats) Analysis

**Figure 1:**
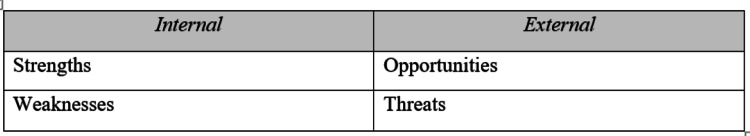
SWOT analysis' internal and external factors

SWOT analysis efficiently identifies an organization's or a project's strengths and weaknesses (internal indicators) with its environment's opportunities and threats (external indicators). Strengths represent the forces of the organization. Additionally, weaknesses represent negative impacts that can influence the product's added value or the quality of the service. Opportunities are assets that an organization can use to its advantage. Moreover, threats are all inappropriate external events that can harm the environment. Figure 1 summarises the indicators used in the SWOT analysis. Owing to the results of the internal and external diagnoses, the SWOT model enables strategies that highlight success factors and eliminate negative impacts.

## Results

SWOT analysis finding

Several internal and external factors impacted the implementation of the EMR in the department. We detected these factors in the initial stages of the system's deployment. They are identified as in Table 1.

**Table 1:** SWOT analysis resulted from the deployment of an EMR in the nephrology department of Hassan II University Hospital in Fez

SWOT ANALYSIS FINDINGS	
Strengths: The implementation of the EMR responds to specifications drawn up in collaboration with the nephrology department (the users of the system). Fluid and continuous communication between users and the EMR project manager. User involvement and commitment during all phases of system implementation. Fast, secure, and immediate access to information. Ergonomic and easy-to-use interface. Responsive web application hosted in a secure environment. Training in the use of the software. Documentation is made available to healthcare professionals. Remote assistance for users. Low financial requirements for EMR implementation. Creation of a database of dialysis patients in the department. Reduction of the risk of data loss thanks to frequent backups of the database. Minimization of the risk of medical errors. Traceability of medical acts and care.	Opportunities: Possible evolution of the EMR's various functionalities. Deployment of the EMR in the different dialysis centers of the Fez-Meknes region and centralization of data in the nephrology department of the Hassan II University Hospital in Fez. Elaboration of statistical studies and longitudinal research thanks to the saved data. Setting up performance indicators from the collected data to establish an effective plan for the improvement of patient care in the different dialysis centers of the region. Interoperability of the current EMR with other health information systems. The growing importance of digital medical records, especially after COVID-19.
Weaknesses: Lack of logistical resources at the institution (computers, unstable network). Loss of time during implementation due to minimal access to the institution's network. Users change weekly. Lack of experienced users of the computer tool. The reluctance of some users due to a lack of time or a fear of reduced productivity for patients. Lack of a long-term maintenance and training plan for the application.	Threats: Recurring network problems. Individual resistance to change management. Users' lack of awareness about the benefits of EMR. Abandonment of the system used by new users. Cyberattacks.

Through the SWOT analysis, several strategies have been considered to leverage different resources, minimize threats, and seize opportunities. The methods presented below stem from the analysis of internal factors in relation to the opportunities and threats identified.

Internal Factors and Opportunities: SO (Strengths, Opportunities)

This strategy focuses on utilizing strengths to capitalize on opportunities. Healthcare professionals from the department have engaged in all stages of EMR implementation, from drafting specifications to user testing the application. This collaboration has led to the development of an application that not only meets the establishment's current needs but also accommodates potential future enhancements. A key planned evolution for the application is the introduction of features tailored to the specific requirements of chronic hemodialysis centers in the region, with the aim of deploying EKILI across these centers. Additionally, through the use of SAAS technology, all collected data can be centralized and securely shared with the nephrology department of Hassan II University Hospital, Fez. The department is then able to process and analyze these data to conduct statistical studies and monitor performance indicators, facilitating the formulation of actionable plans to enhance care quality and patient management.

Internal Factors and Opportunities: WO (Weaknesses, Opportunities)

This strategy seeks to address weaknesses by exploiting opportunities. The COVID crisis has highlighted the need for improvements within the Moroccan healthcare system, making the acceleration of digital transformation across health sectors a priority. In April 2022, several governmental institutions released "Le livre blanc sur la e-santé au Maroc," a white paper on e-health in Morocco [3], outlining the health sector's realities, challenges, and development opportunities. This initiative aims to equip the digital health transformation in the kingdom with adequate human and logistical resources. The digitalization of the health system is a main focus of the National Health System Reform, evidenced by a draft framework law adopted on July 13, 2022, by the Council of Ministers. Concurrently, physicians increasingly recognize the EMR's vital role and are more engaged in its use. Moreover, the service plans to establish a training program for future users and ensure technical maintenance of the application.

Internal Factors and Threats: ST (Strengths, Threats)

The system adheres to the specifications set by the nephrology department, designed to meet user needs and prevent any rejection. Application hosting is secured, with regular database backups to guard against data loss from cyberattacks. Resistance to change is a natural part of digital transformation projects but is not insurmountable. A change management plan has been implemented to involve users in the project, supporting them from the training phase to the autonomous use of the EMR.

Internal Factors and Threats: WT (Weaknesses, Threats)

This strategy aims to safeguard the environment from weaknesses and threats as much as possible. The department's management's strong commitment has increased healthcare providers' awareness of the EMR's importance for both patients and the department. Despite ongoing network issues within the service, the hospital's IT department is actively seeking solutions to improve connectivity throughout the hospital's services. Applications are now accessible via mobile data or alternative internet connections, and the department has upgraded outdated equipment with new ones.

## Discussion

Implementing a computerized medical file offers swift and straightforward access to medical information, representing a significant asset for enhancing care coordination and daily management. The commitment and participation of the department's health professionals in this project are among the most important assets. Indeed, this makes it possible to set up an HIS that perfectly meets the users' needs. According to Jaillah et al. [4], software developed without understanding stakeholders' demands can hinder the implementation of the EHR system. Moreover, a study conducted by the Office of the National Coordinator for Health Information Technology of the U.S. Department of Health and Human Services [5] supports the importance of user engagement in successfully implementing EMRs in healthcare institutions. The same study states that the ergonomic design of the interface is an essential element that helps physicians get used to it. The usability of the application and the possible exploitation of the collected database are strengths of our internal environment. Based on a study undertaken in two university hospitals in Amsterdam conducted by Joukes et al. [6], database exploitation is one of the main strengths of an HIS. Additionally, the EMR has been implemented as part of a PhD study. It is not a profitable project. Thus, it allowed the department to overcome the financial barriers to its implementation. Many studies, including those by Maldonado et al. [7], Mohamed [8], and Bahiru et al. [9], identified a lack of financial resources and funding limitations as significant obstacles to EMR implementation. A deficiency in logistical resources, such as efficient equipment and a stable network, represented a weakness in the internal environment. This challenge has been acknowledged in various studies, including those by Jaillah et al. [4] and Nurvita et al. [10]. User resistance and fear emerged as significant weaknesses, as highlighted in the same studies. These vulnerabilities were observed during the department's application test. Healthcare professionals expressed concerns about the potential loss of patient contact and perceived decreased productivity when using an EMR. Some physicians were apprehensive about the changes associated with the dematerialization of care, which could alter their daily practices - a weakness also identified in Aziz et al.'s [11] study. Additionally, the need for IT-experienced human resources for effective EMR utilization was emphasized in these studies [11]. Consequently, user training becomes a necessary requirement, as highlighted in Ludwick et al.'s [12] study. As shown in the study conducted by Shahmoradi et al. [13], secure medical information exchanges are a significant opportunity for the EMR. Nephrologists could have appropriate access to their patients' medical records. Thus, it allows better coordination of the medical pathway for hemodialysis patients and improves their care. In addition, owing to database statistics, the department could monitor many indicators to improve the management of hemodialysis centers. Kolko et al. provided a set of indicators treated by dialysis [14]. These opportunities can minimize system weaknesses and avoid threats. Numerous research findings, including those by Benedictis et al. [15] and Pereira et al. [16], affirm that the implementation of EMRs enhances healthcare quality, diminishes medical errors, and streamlines physicians' daily workflows. Despite these advantages, there is notable resistance to its adoption among many users. The EMR deployed is a web service application. It is hosted in a secure environment that is resistant to cyberattacks. Hosting healthcare data in the cloud benefits HIS adoption and respects cloud security standards.

Study limitations

The SWOT analysis conducted in this study has enabled the department to pinpoint various internal and external factors that influence the implementation of an HIS. Additionally, it has helped in formulating strategies to capitalize on project strengths and opportunities, mitigate weaknesses, and neutralize threats. Nevertheless, it is critical to acknowledge the limitations of this analysis. Its static nature means the identified factors and recommended strategies may not stay relevant for future HIS deployments due to the evolving environment and changing conditions of subsequent projects. Moreover, SWOT analysis lacks a formal mechanism for prioritizing the identified factors, which could lead to subjective prioritization. Hence, interpretations could be swayed by the personal biases of the individuals conducting the analysis. To mitigate these limitations, it is advised to approach the solutions suggested by the analysis with caution. Enhancing the SWOT analysis with other methodologies, such as benchmarking (already applied in this study) and PESTEL analysis, could be advantageous. Incorporating these methods would enable a more thorough identification of opportunities and threats, offering a more comprehensive perspective to inform strategic decisions regarding the implementation of HISs.

## Conclusions

This SWOT analysis aims to examine the strengths and weaknesses impacting the implementation of EMRs for hemodialysis patients in the Nephrology Department of Hassan II University Hospital. The study is of significant importance to the nephrology network, highlighting various internal and external factors that can influence the digital transformation of hemodialysis records. The implementation of the current EMR system has facilitated improved management of daily workflows, enabling physicians to provide enhanced care to their patients. The suggestions offered can be leveraged to digitize healthcare services across multiple dialysis centers in the Fez-Meknes region. These strategies have culminated in the creation of a feasible and substantial action plan to ensure the successful deployment of EMR within the region. After one year of utilizing EKILI, the EMR system managed more than 453 patient records and over 2,400 prescriptions and hemodialysis sessions. These data will be analyzed to monitor the performance indicators of the department's acute hemodialysis activity.
